# Chorea, psychosis, acanthocytosis, and prolonged survival associated with *ELAC2* mutations

**DOI:** 10.1212/WNL.0000000000006320

**Published:** 2018-10-09

**Authors:** Martin Paucar, Aleksandra Pajak, Christoph Freyer, Åsa Bergendal, Margit Döry, José Miguel Laffita-Mesa, Henrik Stranneheim, Kristina Lagerstedt-Robinson, Irina Savitcheva, Ruth H. Walker, Anna Wedell, Anna Wredenberg, Per Svenningsson

**Affiliations:** From Karolinska University Hospital (M.P., A.P., C.F., M.D., J.L.-M., H.S., K.L., I.S., A. Wedell, A. Wredenberg, P.S.); Karolinska Institutet (M.P., A.P., C.F., Å.B., K.L., A. Wedell, A. Wredenberg, P.S.), Stockholm, Sweden; James J. Peters Veterans Medical Affair Center (R.H.W.), Bronx; and Mount Sinai School of Medicine (R.H.W.), New York, NY.

Biallelic mutations in the elaC ribonuclease Z 2 (*ELAC2*) gene cause a rare mitochondrial disease, the main features of which are hypertrophic cardiomyopathy, delayed psychomotor development, and usually death during childhood.^1^ Only 20 families have been reported with this syndrome.^1–3^ Neither movement disorders nor psychotic features have been described as part of the spectrum of *ELAC2* mutations. We describe a patient with a complex hyperkinetic syndrome and acanthocytosis, harboring biallelic *ELAC2* mutations.

## Case description

An Assyrian 69-year-old woman born in Turkey was evaluated at our center for a slowly progressive Huntingtonian disorder. Family history was negative. The patient came to Sweden at age 33; she never went to school and learned only a few Swedish words, and could perform simple transactions. She had type 2 diabetes mellitus, bilateral sensorineural hearing loss requiring aids since age 58, and a follicular thyroid tumor, and is a silent carrier of α-thalassemia (table e-1; doi.org/10.5061/dryad.j6s420k).

At age 52 years, the patient developed short memory impairment and increasing difficulties managing activities of daily living. Four years later, she reported olfactory hallucinations and became obsessed with cleaning and doing laundry to remove the perceived foul smells. Perioral movements were documented during an emergency room visit at age 56 for psychiatric issues, and were noted by her family to have been present for many years, predating treatment with aripiprazole. The olfactory hallucinations became so severe that she tried to commit suicide by setting her apartment on fire at age 58. This resulted in admission to a psychiatric unit for a year and treatment with aripiprazole. At age 61, worsened gait, balance, and involuntary movements were evident. For the last 3 years, she has required a walker and reports numbness in her calves. She has lost weight, but there is no evidence of feeding dystonia or dysphagia. Recently, she has become fecally incontinent.

On examination, the patient had chorea in the feet and perioral area, dystonic posturing in the hands and reduced arm movements, a waddling gait, bradykinesia, apraxia, and atrophy of the hand muscles (video 1). A simplified psychometric evaluation demonstrated significant deficits in several domains (table e-2). EEG performed twice was normal; neurophysiologic studies revealed a sensorimotor demyelinating polyneuropathy and myopathy. Muscle biopsy revealed both cytochrome oxidase (COX)–negative and ragged-red fibers. Brain MRI demonstrated atrophy in the left perirolandic frontal and temporal cortex, while FDG-PET showed reduced bilateral hypometabolism in these regions and in the insular cortex, more pronounced on the left side (figures e-1 and e-2; doi.org/10.5061/dryad.j6s420k). An incidental aneurysm in the anterior communicating artery was found and treated conservatively; there was no evidence of brain calcifications on CT scans. Huntington disease (HD) and other HD phenocopies were ruled out (appendix; doi.org/10.5061/dryad.j6s420k). Acanthocytes were found on wet blood smears (figure e-3; doi.org/10.5061/dryad.j6s420k). Whole exome sequencing revealed compound heterozygous variants c.394G>A (p.Gly132Arg) and c.1040C>T (p.Ser347Phe) in the *ELAC2* gene (figure e-4; doi.org/10.5061/dryad.j6s420k). ECG was normal but repeated echocardiography demonstrated stable mild ventricular septum hypertrophy without evidence of pump failure. Although we did not find any evidence of respiratory chain defect in the muscle biopsy, the patient's fibroblasts revealed accumulation of unprocessed mitochondrial transcripts but normal steady-state levels of mitochondrial mRNAs and tRNAs (figure 1, A and B). These fibroblasts showed severe growth impairment when using galactose as energy source (figure 1C). Treatment with coenzyme Q10 has been started and regular echocardiography is planned.

10.1212/006320_Video_1 Video 1 The patient has mild chorea involving the lower face, trunk, and upper and lower limbs, which increases with activation. There is dystonic posturing of the fingers and arms, and a mild postural tremor of the right hand. There is bradykinesia and clumsiness of alternating movements of both upper and lower extremities, which has progressed between examinations (at ages 63 and 67 years). She has a waddling, wide-based, gait, which has progressed between the 2 recordings, and the right arm is held in an abducted, dystonic posture. Speech is mildly dysarthric.Download Supplementary Video 1 via http://dx.doi.org/10.1212/006320_Video_1

**Figure:**
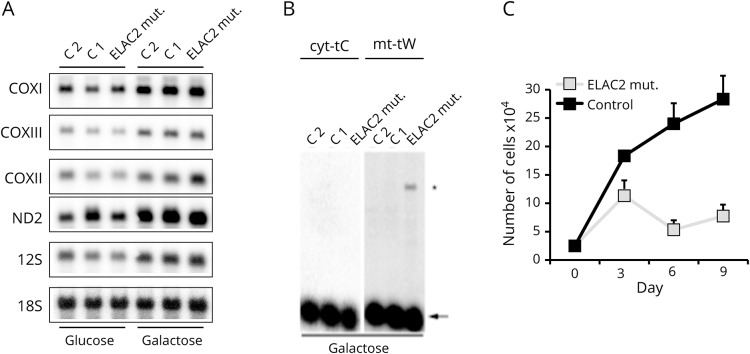
Northern blot analysis of total RNA extractions from primary fibroblasts (A) Steady-state levels of mitochondrial mRNAs. 18S rRNA was used as loading control. Controls: C1 and C2, patient: ELAC2mut. (B) Steady-state levels of mitochondrial tRNA-tryptophane (mt-tW) and the nuclear-encoded tRNA-cysteine (cyto-tC) from control (C1, C2) and patient fibroblasts, grown for 14 days on galactose-containing medium. *Unprocessed mitochondrial transcript, arrow = tRNA. (C) Growth curves of control and patient fibroblasts grown on galactose. Experiments were performed in triplicates. Error bars indicate standard error.

## Discussion

This case demonstrates that *ELAC2* mutations are not exclusively confined to a lethal pediatric cardiomyopathy but can also be associated with symptoms and laboratory findings suggestive of a neuroacanthocytosis syndrome. Most patients with biallelic *ELAC2* mutations reported to date have died during early infancy. In a Saudi cohort of 16 patients harboring the homozygous c.460T>C (p.Phe154Leu) mutation, the mean survival was 4 months.^3^ This dire prognosis makes the prolonged survival of our case remarkable. Apart from our patient, the oldest surviving patient is a 19-year-old patient with severe developmental delay and inability to walk.^2^ There was no dysmorphism or microcephaly, as have been reported with *ELAC2* mutations.^1,2^ Accumulation of unprocessed mitochondrial transcripts and impaired fibroblast growth on galactose support pathogenicity for the *ELAC2* variants we report. Other mitochondrial diseases associated with chorea include Leigh syndrome and mitochondrial myopathy, encephalopathy, lactic acidosis, and stroke-like episodes (MELAS),^4,5^ although these are typically associated with basal ganglia lesions on imaging. Our patient had myopathy, hearing loss, diabetes, and polyneuropathy, which are typical of mitochondrial disease. Even though perioral chorea antedated treatment with aripiprazole, it is not possible to rule out that bradykinesia, dystonic hand posturing, and chorea in the trunk and feet may be side effects of this treatment. The radiologic abnormalities (unilateral frontal and temporal atrophy) and bilateral hypometabolism in the operculum and insula are also new findings for *ELAC2* mutations. Interestingly, injuries in the insula have been reported in association with olfactory hallucinations.^6^

The presence of acanthocytosis, in addition to myopathy, polyneuropathy, and chorea, suggests a neuroacanthocytosis syndrome (i.e., chorea–acanthocytosis and McLeod syndrome, which were excluded in this case); dilated, but not hypertrophic, cardiomyopathy is typical in McLeod syndrome.^7^ One case of MELAS has been reported to be associated with acanthocytosis.^8^ We propose that *ELAC2* should be added to the list of potential causes of neuroacanthocytosis syndromes.

## Publication history

Previously published at bioRxiv doi: 10.1101/244848. Received by *Neurology: Neuroimmunology & Neuroinflammation* February 8, 2018. Accepted in final form July 19, 2018.
